# A failure in decryption process for bivariate polynomial reconstruction problem cryptosystem

**DOI:** 10.1016/j.heliyon.2024.e25470

**Published:** 2024-02-01

**Authors:** Siti Nabilah Yusof, Muhammad Rezal Kamel Ariffin, Sook-Chin Yip, Terry Shue Chien Lau, Zahari Mahad, Ji-Jian Chin, Choo-Yee Ting

**Affiliations:** a Institute for Mathematical Research, Universiti Putra Malaysia, 43400 UPM Serdang, Selangor, Malaysia; b Department of Mathematics and Statistics, Faculty of Science, Universiti Putra Malaysia, Selangor, Malaysia; c Faculty of Engineering, Multimedia University, Cyberjaya 63100, Selangor, Malaysia; d Faculty of Computing and Informatics, Multimedia University, Cyberjaya 63100, Selangor, Malaysia; e School of Engineering, Computing and Mathematics (Faculty of Science and Engineering), University of Plymouth, Drake Circus, Plymouth PL 48AA, UK

**Keywords:** Polynomial reconstruction problem, Post-quantum cryptography, Decryption failure, Univariate polynomial, Bivariate polynomial

## Abstract

In 1999, the Polynomial Reconstruction Problem (PRP) was put forward as a new hard mathematics problem. A univariate PRP scheme by Augot and Finiasz was introduced at Eurocrypt in 2003, and this cryptosystem was fully cryptanalyzed in 2004. In 2013, a bivariate PRP cryptosystem was developed, which is a modified version of Augot and Finiasz's original work. This study describes a decryption failure that can occur in both cryptosystems. We demonstrate that when the error has a weight greater than the number of monomials in a secret polynomial, *p*, decryption failure can occur. The result of this study also determines the upper bound that should be applied to avoid decryption failure.

## 1 Introduction

A valid and secure cryptosystem can be designed using a good hard mathematical problem in cryptography. Cryptography is an important mechanism in data security where the cryptography algorithm makes communication possible in the presence of an adversary [8], [30]. User's private data in embedded system needs to be protected and authenticated. It is essential for users to ensure that data consumed is valid [10], [28]. Shor's algorithm has successfully solved classical problems such as the integer factorization problem (IFP) and the discrete logarithm problem (DLP) in polynomial time, where a quantum computer can attack cryptosystems that rely on such difficult mathematical problems [1], [3], [34]. Among the well-known cryptographic schemes that are algorithmically insecure in post quantum cryptography are RSA, El-Gamal, and Elliptic Curve Cryptosystem [7], [22]. The National Institute of Standards and Technology (NIST) has called for a search for quantum-resistant algorithms [4], [11], [13], [33].

Hence, this shows that post-quantum cryptography is preferable for information security purposes. Post-quantum cryptography is a cryptographic algorithm that is believed to be secure from the attack of quantum computer [21]. Post-quantum cryptography also consists of five major types which are lattice-based, code-based, isogeny-based, hash-based and multivariate-based cryptography [9], [19]. The Quantum Algorithm Zoo website lists useful hard mathematical problems that may be immune to a quantum computing attack [20]. Thus, cryptographers must investigate diverse hard problems so that the new design cryptosystems are safe from the attack of quantum computers [18]. The evaluation of time intricacy and memory space for the attack is to ensure and validate the safety of the cryptosystems [26], [27].

Quantum Algorithm Zoo introduced the PRP as a difficult mathematical problem in post-quantum cryptography [20]. This problem was introduced in 1999 when PRP developed a formulation equivalent to Reed-Solomon error-correcting codes [6], [29], [31]. The problem also contains the full intricacy against the quantum computers with the complexity of O(q) in which *q* contains *n* bits of prime. Besides that, a wide range of research on the PRP has been conducted based on the solvability and robustness [25].

This problem can be easily solved if the error's weight, *w*, is at most $\frac{n-k}{2}$. The parameter *n* represents the number of elements of the vector, while the parameter *k* represents the polynomial degree. This equation has been upgraded into $w\leq n-\sqrt{kn}$ [16]. Augot and Finiasz suggested a univariate PRP cryptosystem in 2003, where we call this scheme the AF-Cryptosystem. A univariate polynomial is used in the AF-Cryptosystem [23], [24]. The AF-Cryptosystem also applied two PRP types: the first PRP is defined in [20], and the second PRP is built to guarantee the process of decryption. The second PRP is denoted as the Augot and Finiasz Solvable PRP (AF-SPRP), which is described below:

Definition 1 ***(Augot and Finiasz Solvable PRP)*** Given n, k, t and ${\left(x_{i},y_{i}\right)}_{i=1,\cdots ,n}$, output any polynomial p such that deg<k and $p(x_{i})=y_{i}$ for at least t values of i where t=n−w.

From Definition 1, the decryption process can occur in the AF-Cryptosystem. From the Cartesian plane, if we obtain *t* points, a polynomial is required to be yielded in which this polynomial consists of all the points where *t* is the zero element in a vector. The decryption process in the AF-Cryptosystem can be done using Lagrange interpolation.

Nevertheless, the AF-Cryptosystem was managed to be fully cryptanalyzed by Coron [12]. Next, a bivariate PRP cryptosystem was proposed in 2013 by Ajeena et al.; this cryptosystem is called the AAK-Cryptosystem [2]. The AAK-Cryptosystem is the modified version of the AF-Cryptosystem where they used bivariate polynomial and Vandermonde method. The creators of AAK-Cryptosystem mentioned that if the amount of variables increases, then the cryptosystem's security level can be improved.

**Our contribution.** In this paper, we analyze the decryption process for both cryptosystems, which is different from our published papers in [36], [37]. In our published papers, we put forward results that discusses the AAK-Cryptosystem is not indistinguishable chosen plaintext attack (IND-CPA) secure and how to retrieve the private key from the AAK-Cryptosystem. While our findings in this paper indicate that decryption errors can occur in both cryptosystems if the weight of the big error vector *E* is greater than the number of monomials in the secret polynomial *p*.

**Organization of the article.** This paper's setup is as follows: in Section 2, we put forward the fundamentals of PRP, Lagrange interpolation, and Vandermonde method and outline both AF-Cryptosystem and AAK-Cryptosystem. In Section 3, we explain our propositions for decryption failure in both cryptosystems and give an example for this analysis. Finally, we discuss our result in Section 4 and we conclude our findings in Section 5.

## 2 Materials and methods

This section explains the fundamental knowledge about PRP, Lagrange interpolation, Vandermonde method, AF-Cryptosystem and AAK-Cryptosystem.

### 2.1 PRP

The PRP is known since the generalized Reed-Solomon list decoding problem has been reduced to it [32]. Next, we describe PRP based on [20], which shown down below:

Definition 2 ***(PRP from Quantum Zoo)*** Let $p(x)=a_{k}x^{k}+\cdots +a_{1}x+a_{0}$ be a polynomial over finite field $F_{q}$. One is given access to the oracle and query value of $x_{i}\in F_{q}$ where 1≤i≤k+1 then output coefficients $a_{k},\ldots ,a_{0}$ to determine p(x).

From Definition 2, this shows that when the oracle input $x\in F_{q}$, then it will output p(x). Then, this provides us the coefficients $a_{k},\ldots ,a_{0}$ [20]. Classically, we need k+1 queries to identify the number of coefficients. Therefore, the query complexity in PRP for a univariate polynomial with a degree equal to *k* is $O\left(\begin{array}{c} k+1\\ k \end{array}\right)$.

### 2.2 Computational complexity of PRP

We know that p(x) has a degree equal to *k*, and p(x) contains k+1 coefficients, equivalent to q−1, hence k=q−2. Thus,

$$O\left(\begin{array}{c} k+1\\ k \end{array}\right)=O(q-1).$$

It is impractical for us to query input *x* if $q\approx 2^{n}$ is exponentially large. This shows that solving PRP would take exponential time, which is $O(2^{n})$.

### 2.3 Lagrange interpolation

The Lagrange interpolation method can identify a polynomial based on the observed value at each observed point. Besides that, Lagrange interpolation is regularly utilized in cryptography to share secret and coding computing [14]. The Lagrange interpolation is where we are provided *n* real values $x_{1},x_{2},x_{3}\ldots ,x_{n}$ and $y_{1},y_{2},y_{3}\ldots ,y_{n}$, then output a polynomial *p* that contains real coefficients which satisfies $p(x_{i})=y_{i}$ where i=1,2,3,…,n [17]. Polynomial *p* must have a degree less than the real values where degree(p)<n. The Lagrange interpolation formula with *n*th order is as follows,

$$f(x)=\frac{\left(x-x_{1})(x-x_{2})\ldots (x-x_{n}\right)}{\left(x_{0}-x_{1})(x_{0}-x_{2})\ldots (x_{0}-x_{n}\right)}\times y_{0}+\frac{\left(x-x_{0})(x-x_{2})\ldots (x-x_{n}\right)}{\left(x_{1}-x_{0})(x_{1}-x_{2})\ldots (x_{1}-x_{n}\right)}\;\;\;\;\times y_{1}+\ldots +\frac{\left(x-x_{0})(x-x_{1})\ldots (x-x_{n-1}\right)}{\left(x_{n}-x_{0})(x_{n}-x_{1})\ldots (x_{n}-x_{n-1}\right)}\times y_{n}.$$

The AF-Cryptosystem utilized Lagrange interpolation in decryption process.

### 2.4 Vandermonde method

An interpolation polynomial with two or more dimensions is determined using the Vandermonde method. Given points that have two variables where $\left(x_{1},y_{1}),(x_{2},y_{2}),\ldots ,(x_{n},y_{n}\right)$, for each point, one must obtain the polynomial values $z_{1},z_{2},\ldots ,z_{n}$, correspondingly. The two variables polynomial with the degree of n−1 can be obtained by using the following steps,

1. Formulate the formula of a polynomial with the degree n−1 where this polynomial contains two variables.
2. Calculate the polynomial at the given points.
3. Solve the system of linear equations.

The problem can be presented in the form V⋅c=Z where *V* is a Vandermonde matrix with the dimension of n×n, also known as coefficients matrix [15], [35]. Parameter *Z* contains *z* values, while parameter *c* is the coefficient vector. The AAK-Cryptosystem applied the Vandermonde method in the decryption process.

### 2.5 AF-cryptosystem

Augot and Finiasz introduced a univariate PRP cryptosystem which describes down below [5]. Considering that *n* is the number of elements in the vector and the AF-cryptosystem applied the following parameters in Table 1.

**Table 1:** Parameters.

Parameter	Remark
$F_{q}$	A finite field of size *q*
*n*	The number of elements in the vector
*k*	Its dimension
*W*	The weight of big error vector, *E* where PRP is hard when, $W>\frac{n-k}{2}$[2]
*w*	The weight of small error vector, *e* that enabling the PRP to decrypt the ciphertext when $w\leq \frac{n-k}{2}$[12]

Remark 1 The parameter *w* is the vector's maximum number of nonzero elements.

Remark 2 The parameter n−w is known as the number of zero elements of the vector.

The proposed AF-Cryptosystem is as follows:

**Algorithm 1:**
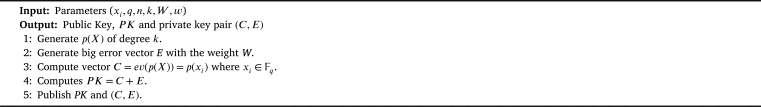
Key generation process.

**Algorithm 2:**
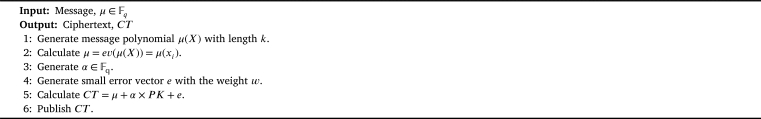
Encryption process.

**Algorithm 3:**
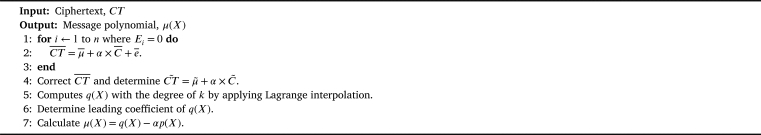
Decryption process.

#### 2.5.1 Proof of correctness

Proposition 1 *The message polynomial* μ(x) *can be obtained through the decryption algorithm in AF-Cryptosystem.*

Proof Refer to Appendix A. □

### 2.6 AAK-cryptosystem

The AF-Cryptosystem was altered by Ajeena et al. to create the bivariate PRP cryptosystem that is described below [2]. The AAK-Cryptosystem applied the parameters in Table 1. The proposed modified cryptosystem is as follows:

**Algorithm 4:**
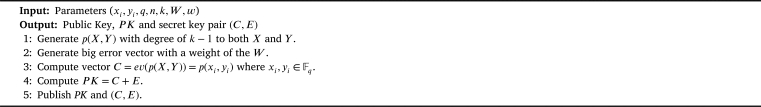
Key generation process.

**Algorithm 5:**
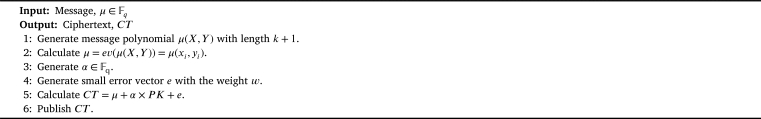
Encryption process.

**Algorithm 6:**
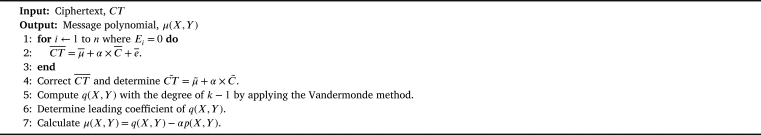
Decryption process.

#### 2.6.1 Proof of correctness

Proposition 2 *The proof of the decryption algorithm in AAK-Cryptosystem is correct.*

Proof Refer to Appendix B. □

## 3 The decryption failure

This section explains how decryption failure can occur in the AF-Cryptosystem and AAK-Cryptosystem. A numerical illustration is also provided.

### 3.1 Decryption failure in AF-cryptosystem

Proposition 3 *If the weight of nonzero element in big error E is* W>k+1 *, then the decryption process in AF-Cryptosystem cannot occur.*

Proof Refer to Appendix C. □

#### 3.1.1 Numerical illustration for Proposition 3

In this section, inline with Proposition 3, we put forward a numerical example where the system owner incorrectly sets the system parameters such that n−W<k+1 which would lead to the system owner unable to decrypt the ciphertext.

Example 1 Let n=7, k=2, w=2 and W=5 in $F_{11}$. Given x=(9,8,7,6,5,4,3). We start with the key generation process by taking the private polynomial,

$$p(x)=x^{2}+2x+6$$

and big error vector *E*,

E=(1,2,3,4,5,0,0).

The public key is:

PK=C+E.

Vector *C* is obtained by the evaluation of p(x) where:

p(9)=6,p(8)=9,p(7)=3,p(6)=10,

p(5)=8,p(4)=8,p(3)=10.

Hence, C=(6,9,3,10,8,8,10). Then, compute *PK* as follows,

PK=C+E=(6,9,3,10,8,8,10)+(1,2,3,4,5,0,0)=(7,0,6,3,2,8,10).

Next, in encryption process, we evaluate μ(x)=x+5 codeword *μ* which shown as follows,

μ(9)=3,μ(8)=2,μ(7)=1,μ(6)=0,

μ(5)=10,μ(4)=9,μ(3)=8.

Thus, we have

μ=(3,2,1,0,10,9,8).

Next, we generate a constant $\alpha =2\in F_{11}$ and a small error vector, *e* where

e=(6,7,0,0,0,0,0).

Observe that the weight for the small error vector is w=2. Then, the *CT* is:

CT=μ+α×PK+e=(3,2,1,0,10,9,8)+2×(7,0,6,3,2,8,10)+(6,7,0,0,0,0,0)=(3,2,1,0,10,9,8)+(3,0,1,6,4,5,9)+(6,7,0,0,0,0,0)=(1,9,2,6,3,3,6).

In the decryption process, based on the AF-Cryptosystem, we need to consider the position of zero elements in *E* where n−W=2. From *E*, we have

$$E_{6}=E_{7}=0.$$

Therefore, we obtain two shadows $\bar{CT}=CT_{6}=CT_{7}=(3,6)$. Next, Lagrange interpolation is applied to find q(x). The degree of polynomial q(x) must be k=2. From $\bar{CT}$, we have

q(4)=3andq(3)=6

Hence, the unique polynomial q(x) is as follows,

$$q(x)=\frac{\left(x-x_{7}\right)}{\left(x_{6}-x_{7}\right)}(CT_{6})+\frac{\left(x-x_{6}\right)}{\left(x_{7}-x_{6}\right)}(CT_{7})=\frac{\left(x-3\right)}{\left(4-3\right)}(3)+\frac{\left(x-4\right)}{\left(3-4\right)}(6)=3x-9-6x+24\;\text{mod}\;11=8x+4.$$

As we can see here, q(x) has a degree of 1, which is smaller than p(x). Therefore, we cannot identify μ(x) due to the small size of q(x). Hence, the decryption process is a failure.

### 3.2 Decryption failure in AAK-cryptosystem

This section presents the scenario where the decryption process in AAK-Cryptosystem is a failure. A larger size of *W*, will make it difficult to determine the message polynomial, μ(x,y).

Proposition 4 *If the weight of nonzero element in big error E is larger than number of monomial of secret polynomial* p(x,y) *, then the decryption process in AAK-Cryptosystem cannot occur.*

Proof Refer to Appendix D. □

#### 3.2.1 Numerical illustration of Proposition 4

In this section, inline with Proposition 4, we put forward a numerical example where the system owner incorrectly sets the system parameters such that $n-W<k^{2}$ which would lead to the system owner unable to decrypt the ciphertext.

Example 2 Let n=10, k=2, w=1 and W=7 in $F_{11}$. Given x=(4,3,2,1,2,1,2,3,4,3) and y=(1,1,2,2,3,3,4,4,3,0). We start with the key generation process by taking the secret polynomial,

p(x,y)=xy+2x+y+1

and big error vector *E*,

E=(1,2,3,4,1,2,3,0,0,0).

The public key is:

PK=C+E.

Vector *C* is obtained where the p(x,y) is evaluated down below:

p(4,1)=3,p(3,1)=0,p(2,2)=0,p(1,2)=7,p(2,3)=3,

p(1,3)=9,p(2,4)=6,p(3,4)=1,p(4,3)=2,p(3,0)=7.

Thus, C=(3,0,0,7,3,9,6,1,2,7). Then, the *PK* is as follows,

PK=C+E=(3,0,0,7,3,9,6,1,2,7)+(1,2,3,4,1,2,3,0,0,0)=(4,2,3,0,4,0,9,1,2,7).

Next, in encryption process, we evaluate μ(x,y)=2x+4y+5 codeword *μ* which shown as follows,

μ(4,1)=4,μ(3,1)=2,μ(2,2)=4,μ(1,2)=2,μ(2,3)=8,

μ(1,3)=6,μ(2,4)=1,μ(3,4)=3,μ(4,3)=1,μ(3,0)=9.

Hence, we obtain

μ=(4,2,4,2,8,6,1,3,1,9).

Next, we generate a secret value $\alpha =3\in F_{11}$ and a small error vector, *e* such that

e=(2,0,0,0,0,0,0,0,0,0).

Observe that the weight for the small error vector is w=1. Then, *CT* is:

CT=μ+α×PK+e=(4,2,4,2,8,6,1,3,1,9)+3×(4,2,3,0,4,0,9,1,2,7)+(2,0,0,0,0,0,0,0,0,0)=(4,2,4,2,8,6,1,3,1,9)+(1,6,9,0,1,0,5,3,6,10)+(2,0,0,0,0,0,0,0,0,0)=(7,8,2,2,9,6,6,6,7,8).

In the decryption process, based on the AAK-cryptosystem, we need to consider the position of zero elements in *E* where n−W=3. From *E* we have

$$E_{8}=E_{9}=E_{10}=0.$$

Thus, we contain three shadows $\bar{CT}=CT_{8}=CT_{9}=CT_{10}=(6,7,8)$. The next step is to find a unique polynomial q(x,y) using the Vandermonde method. Polynomial q(x,y) must be with the degree of k−1=1 for *X* and *Y*. Let $q(X,Y)=q_{1}xy+q_{2}x+q_{3}y+q_{4}$, we have,

$$q(3,4)=q_{1}(1)+q_{2}(3)+q_{3}(4)+q_{4}=6$$

$$q(4,3)=q_{1}(1)+q_{2}(4)+q_{3}(3)+q_{4}=7$$

$$q(3,0)=q_{1}(0)+q_{2}(3)+q_{3}(0)+q_{4}=8.$$

We need to determine the coefficients for q(X,Y) by using Gaussian elimination,

$$\left[\begin{array}{cccc} 1 & 3 & 4 & 1\\ 1 & 4 & 3 & 1\\ 0 & 3 & 0 & 1 \end{array}\right]\left[\begin{array}{c} q_{1}\\ q_{2}\\ q_{3}\\ q_{4} \end{array}\right]=\left[\begin{array}{c} 6\\ 7\\ 8 \end{array}\right].$$

Then, the equation of the system is

$$\left[\begin{array}{cccc} 1 & 0 & 0 & 2\\ 0 & 1 & 0 & 2\\ 0 & 0 & 1 & 2 \end{array}|\;\begin{array}{c} 3\\ 1\\ 0 \end{array}\right].$$

As we can see here, the system shows that $q_{4}$ is a free variable and is not a unique solution. Hence, we cannot identify q(x,y). From $\bar{CT}=(6,7,8)$, we have insufficient information to identify unique polynomial q(x,y). Hence, the decryption process cannot be done.

## 4 Discussion

Based on the results, we need to ensure the weight of big error vector *W*, is less than the number of monomials of the secret polynomial *p*, for both AF-Cryptosystem and AAK-cryptosystem. The users of these cryptosystems need to take into consideration information regarding the boundary value for *W*, to prevent decryption failure from occurring.

## 5 Conclusion

This paper presents that decryption failure can occur in AF-Cryptosystem and AAK-Cryptosystem. When *W* is greater than the number of monomials of secret polynomial *p*, then we cannot determine unique polynomial *q*. Hence, we cannot decrypt the ciphertext, *CT*, to identify the message polynomial, *μ*. Thus, the recommended weight for big error vector, *E* to be used in AF-Cryptosystem and AAK-Cryptosystem are $\frac{n-k}{2}<W\leq k+1$ and $\frac{n-k}{2}<W\leq k^{2}$ respectively so that decryption process can occur. For the future works, we would suggest an investigation into whether the size of the message polynomial that is used in both cryptosystems could also contribute towards decryption failure.

## Availability of data and materials

Not applicable.

## Consent of publication

Not applicable.

## CRediT authorship contribution statement

**Siti Nabilah Yusof:** Writing – original draft, Methodology, Formal analysis, Conceptualization. **Muhammad Rezal Kamel Ariffin:** Writing – review & editing, Validation, Supervision, Funding acquisition. **Sook-Chin Yip:** Writing – review & editing, Funding acquisition. **Terry Shue Chien Lau:** Formal analysis. **Zahari Mahad:** Formal analysis. **Ji-Jian Chin:** Funding acquisition, Formal analysis. **Choo-Yee Ting:** Formal analysis.

## Declaration of Competing Interest

The authors declare that they have no known competing financial interests or personal relationships that could have appeared to influence the work reported in this paper.

## Appendix A The proof of Proposition 1

Proof Based on the ciphertext *CT*, the message polynomial μ(x) can be recovered where:

(A.1) CT=μ+α×PK+e=μ+α×(C+E)+e.

Consider $E_{i}=0$, which is the shortened code of Reed-Solomon code of dimension *k*, $\bar{RS_{k}}$. Given that $\bar{\mu }$, $\bar{C}$, $\bar{e}$ and $\bar{CT}$ are the shortened code for μ,C,e and *C*T respectively then *E* is disappeared. Now, (A.1) turns into

(A.2) $$\bar{CT}=\bar{\mu }+\alpha \times \bar{C}+\bar{e}.$$

From (A.2), $\bar{\mu }+\alpha \times \bar{C}\in \bar{RS_{k}}$. Given that the weight of *e* is smaller than error correction capacity $\bar{RS_{k}}$ then correct $\bar{CT}$ and determine $\overset{˜}{\mu }+\alpha \times \overset{˜}{C}$. By applying Lagrange interpolation, calculate q(x) which has the degree equals to *k* such that

(A.3) $$ev(q(x_{i}))=\overset{˜}{\mu_{i}}+\alpha \times \overset{˜}{C_{i}}$$

for i∈{1,2,…,n}. Since $ev(q(x_{i}))$ is the evaluation of $q(x_{i})$, vector $\overset{˜}{C}=ev(p(x_{i}))$ is the evaluation of $p(x_{i})$ and vector $\overset{˜}{\mu }=ev(\mu (x_{i}))$ is the evaluation of $\mu (x_{i})$ then

(A.4) $$q(x_{i})=\mu (x_{i})+\alpha p(x_{i})\mu (x_{i})=q(x_{i})-\alpha p(x_{i}).$$

Based on (A.4), the μ(x) has been recovered. Thus, the proof is terminated. □

## Appendix B The proof of Proposition 2

Proof Based on the ciphertext *CT*, the message polynomial μ(x,y) can be recovered where:

(B.1) CT=μ+α×PK+e=μ+α×(C+E)+e.

The position $E_{i}=0$ is considered the shortened code of Reed-Solomon of dimension *k*, $\bar{RS_{k}}$. Given that $\bar{\mu }$, $\bar{C}$, $\bar{e}$ and $\bar{CT}$ are the shortened code for μ,C,e and *CT* respectively then *E* is disappeared. Now, (B.1) turns

(B.2) $$\bar{CT}=\bar{\mu }+\alpha \times \bar{C}+\bar{e}.$$

From (B.2), $\bar{\mu }+\alpha \times \bar{C}\in \bar{RS_{k}}$. Given that *e* is with the weight less than error correction capacity $\bar{RS_{k}}$ then correct $\bar{CT}$ and determine $\overset{˜}{\mu }+\alpha \times \overset{˜}{C}$. By applying Vandermonde method, compute q(x,y) which has the degree equals to k−1 where

(B.3) $$ev(q(x_{i},y_{i}))=\overset{˜}{\mu_{i}}+\alpha \times \overset{˜}{C_{i}}$$

for i∈{1,2,…,n}. Since $ev(q(x_{i},y_{i}))$ is the evaluation of $q(x_{i},y_{i})$, vector $\overset{˜}{C}=ev(p(x_{i},y_{i}))$ is the evaluation of $p(x_{i},y_{i})$ and vector $\overset{˜}{\mu }=ev(\mu (x_{i},y_{i}))$ is the evaluation of $\mu (x_{i},y_{i})$, hence

(B.4) $$q(x_{i},y_{i})=\mu (x_{i},y_{i})+\alpha p(x_{i},y_{i})\mu (x_{i},y_{i})=q(x_{i},y_{i})-\alpha p(x_{i},y_{i}).$$

Based on (B.4), the μ(x,y) can be recovered. □

## Appendix C The proof of Proposition 3

Proof Based on Table 1, Alice generates p(x) with a degree equal to *k*, and during the key generation process, *E* with weight *W* is generated by Alice. Given that p(x) be as follows,

$$p(x_{i})=x^{k}+\ldots +a_{2}x^{2}+a_{1}x^{1}+a_{0}.$$

Thus, p(x) has k+1 coefficients. Suppose that Alice chooses the weight *E* as W>k+1. The position of zero and nonzero elements in *E* can vary. The ciphertext, *CT* contains *n* vector elements given by,

$$CT_{i}=\mu_{i}+\alpha \cdot PK_{i}+e_{i}\;\forall \;1\leq i\leq n.$$

In the decryption process, the position of zero elements is important, and there are n−W zero elements. The decryption process will acquire $\bar{CT}$ where $\bar{CT}$ contains n−W elements. Next, the unique polynomial q(x) with degree *k* can be determined by applying Lagrange interpolation. Polynomial q(x) must have k+1 coefficients which are the same as the secret polynomial p(x). If Alice takes W>k+1, it will result in the polynomial q(x), which contains less number of coefficients in $\bar{CT}$. This is because n−W<k+1. From here, by using Lagrange interpolation, the q(x) that will be acquired contains degree n−W−1, which is less than *k*. One must compute q(x)−α⋅p(x) in order to recover μ(x). Since q(x) is smaller than p(x), we will recover μ(x) with a degree less than p(x). Hence, the decryption process is a failure due to the weight of the big error vector, *E*. □

## Appendix D The proof of Proposition 4

Proof Based on the parameters in Table 1, we start with the key generation process, where Alice has a private polynomial p(x,y) of degree k−1 for both *x* and *y* and a big error vector *E* its weight is *W*. Given that p(x,y) is as follows,

$$p(x_{i},y_{i})=x^{k-1}y^{k-1}+\ldots +a_{1,1}x^{1}y^{1}+a_{1,0}x^{1}+a_{0,1}y^{1}+a_{0,0}.$$

Then, p(x,y) has $k^{2}$ coefficients. Suppose that Alice chooses the weight *E* as $W>k^{2}$. The position of zero and nonzero elements in *E* can vary. The ciphertext, *CT* contains *n* vector elements given by,

$$CT_{i}=\mu_{i}+\alpha \cdot PK_{i}+e_{i}\;\forall \;1\leq i\leq n$$

In the decryption process, the position of zero elements is essential, and there are n−W zero elements. The decryption process will obtain $\bar{CT}$, with n−W elements in $\bar{CT}$. Next, by using the Vandermonde method, we can determine q(x,y) with degree k−1 where q(x,y) contains $k^{2}$ coefficients which are the same as the secret polynomial p(x,y). If Alice selects *W* to be more than $k^{2}$, it will result in q(x,y) containing less number of coefficients in $\bar{CT}$. This is because $n-W<k^{2}$. Using the Vandermonde method, the solution we will obtain is not unique. This would lead to a situation where we have to calculate a system of equations where the number of equations is less than the number of variables. Therefore, matrix *V* is not a square matrix. Upon solving such systems, we would arrive at the following situations:

i) infinitely many solutions
ii) no solution

The μ(x,y) can be recovered by calculating q(x,y)−α⋅p(x,y). Since q(x,y) can be i) or ii), then we cannot calculate q(x,y)−α⋅p(x,y). Thus, the decryption process is a failure due to the weight of the big error vector, *E*. □
